# The application of social recommendation algorithm integrating attention model in movie recommendation

**DOI:** 10.1038/s41598-023-43511-1

**Published:** 2023-10-07

**Authors:** Pengjia Cui, Boshi Yin, Baichuan Xu

**Affiliations:** School of Information and Communication Engineering, Communication University of China, Beijing, 100024 China

**Keywords:** Computational science, Computer science

## Abstract

To improve the accuracy of recommendations, alleviate sparse data problems, and mitigate the homogenization of traditional socialized recommendations, a gated recurrent neural network is studied to construct a relevant user preference model to mine user project preferences. Through the Preference Attention Model Based on Social Relations (PASR), this study extracts user social influence preferences, performs preference fusion, and obtains a Recommendation Algorithm Based on User Preference and Social Influence (UPSI). The study demonstrates that the UPSI algorithm outperforms other methods like the SocialMF algorithm, yielding improved recommendation results, higher HR values, and larger NDCG values. Notably, when the K value equals 25 in Top-K recommendation and using the CiaoDVDs dataset, the NDCG value of the UPSI algorithm is 0.267, which is 0.120 higher than the SocialMF algorithm's score. Considering the user's interaction with the project and their social relationships can enhance the effectiveness of recommendations. Unlike other variants, the UPSI algorithm achieves a maximum hit rate HR value of 0.3713 and NDCG value of 0.2108 in the Douban dataset. In the CiaoDVDs dataset, the maximum hit rate HR value of UPSI is 0.4856, 0.0333 higher than UPS-A, 0.0601 higher than UPS, and 0.0901 higher than UP. Research methods can effectively improve the homogenization problem of traditional socialized recommendations, increase algorithm hit rates and NDCG values. Compared to previous studies, research methods can more fully explore the preference correlation between users, making recommended movies more in line with user requirements.

## Introduction

The development of the Internet generates a large amount of information. How can users quickly find the information they need in the vast amount of information data; meanwhile, how can information publishers quickly push information to target users? This is the problem that needs to be solved in the development process of the Internet^1–3^. The emergence of recommendation systems effectively alleviates this problem. Through personalized recommendations from the recommendation system, users receive items that meet their preferences. The recommendation algorithm is the crucial aspect of a recommendation system^4^. Collaborative filtering is a commonly used recommendation algorithm. Although its recommendation effect is good, it shows some limitations in processing massive information. The cold treatment problem caused by new users of new projects cannot be solved by collaborative filtering. Additionally, it does not perform optimally in handling sparse data^5^. Some studies have incorporated user socialization information and other related information into recommendation algorithms to improve sparse user ratings. This can improve the ability to capture user preferences and enable recommendation algorithms in obtaining superior performance. The emergence of socialized recommendations has promoted personalized distribution of online resources, making it easier for users to discover their favorite projects, and is conducive to the collaborative development of multiple disciplines^6,7^. However, there is a problem of homogenization in general social recommendations, which does not take into account the differences in preferences between users and friends under different projects. To address this issue, attention models are applied from the standpoint of user preferences and social influence to construct suitable algorithms for social movie recommendations. This study aims to improve the performance of recommendation algorithms and achieve personalized recommendations. Unlike previous studies, the study considers the different impacts of different friends on users. By introducing an attention mechanism, the attention weights of users towards different friends are obtained. It makes the obtained social influence preferences of users more accurate and facilitates the enhancement of recommendation accuracy.

## Related work

In the Internet, people can carry out entertainment, shopping and other activities, which greatly facilitate people's life. The significant user population has propelled the rapid expansion of information data, causing users to encounter difficulties filtering information. The emergence of recommendation systems solves this problem by providing users with favorite projects. Yu X et al. face the recommendation problem of healthcare wearable devices and classify them into corresponding features based on their relevance to healthcare, and combine them into user features. Integrate relevant feature information through the factorization machine algorithm and recommend relevant results. The results show that the method is effective^8^. Zheng G et al. consider users' malintent ratings in project recommendations. Based on the characteristics of item tags, the related collaborative filtering algorithm is obtained. After verification, the recommendation effect of this method is good^9^. Pan G A et al. have dynamic variability and other factors based on user preferences. On the basis of conversation, attention mechanism is introduced to obtain the representation of user friends, graph neural network is referenced, and relevant social recommendation algorithms are constructed. The method is tested to be effective^10^. Zhao G et al. analyze the internal factors of users in social recommendation comments and learn the weights of relevant factors through attention mechanisms. After verification, considering factors such as user emotional bias when recommending can achieve better recommendation results^11^.

Zhong T et al. develops relevant interest point recommendation algorithms based on graph spacing networks to address the issues of cold start in recommendation algorithms. This algorithm utilizes a multi head attention mechanism to distinguish different preferences of user interest points. The results show that the accuracy of method recommendation is higher^12^. Huang L et al. face the problem of interest point recommendation and model the correlation between random two historical checkins through a self attention mechanism. Verified by the dataset, this method performs better^13^. Jiang N et al. utilize social network information in recommendation algorithms by utilizing social aggregation neural networks based on attention mechanisms to make relevant recommendations. The results show that the application effect of the method is good^14^. When Yj A et al. recommend projects, they choose graph convolutional networks to construct two-level graph attention networks to enhance the effectiveness of social recommendations. The results show that the method is effective^15^. Wu L et al. apply neural models to the recommendation problem and construct the Collaborative Neural Social Recommendation (CNSR) algorithm, which consists of a social embedding part and a collaborative neural recommendation part. After experiments, it was found that the proposed algorithm is effective^16^. He X et al. use the Neural Collaborative Filtering (NCF) algorithm in recommendation systems to learn the interaction function between users and projects through multi-layer perceptrons. The results show that the proposed algorithm has good recommendation performance^17^. Li H et al. propose Bayesian personalized ranking from implicit feedback (BPR) based on implicit feedback to learn the potential feature vectors of users and projects in the face of recommendation problems. The results show that the proposed method has a good recommendation effect^18^. Drif A et al. propose an integrated variational autoencoder recommendation framework for recommendation problems. It specifies a process of converting the predicted utility matrix of secondary recommenders into interest probabilities. From the results, it can be seen that the recommendation effect of this method is good^19^.

In summary, there is a lot of research on social recommendation algorithms in the field of project recommendation. These recommendation algorithms can improve the sparsity of data, but there are still some problems. Different friends have different impacts on target users. When considering user social relationships, treating the impact of friends on users together can compromise the accuracy of recommendation results. Moreover, user preferences are prone to change over time. Some algorithms do not consider the time information of user interaction with the project when predicting the projects that users may like. From the perspective of dynamic changes in user preferences and social relationships, research is conducted on social recommendation algorithms. Given the excellent performance of attention mechanism in weight learning, its application in research can improve recommendation performance.

## Social movie recommendation algorithm based on attention model

### User preference model based on interaction sequences

The development of network technology facilitates people's daily life and entertainment. On the internet, people discover their favorite products, movies, etc. through the use of recommendation systems. However, in previous social recommendations, it was mostly assumed that users' social networks were homogeneous, treating them equally with their friends’ preferences, while neglecting that the preferences of two individuals under the same item may not be exactly the same. Over time, user preferences may undergo changes, along with corresponding modifications to project selection. Modeling user project interaction sequences is beneficial for more accurate characterization of user preferences. It utilizes an attention machine model to capture the social impact preferences of target users when interacting with different projects. This can result in more accurate recommendation results. In this regard, research has used Recommendation Algorithm Based on User Preference and Social Influence (UPSI) to recommend movies. The UPSI algorithm starts from two perspectives: the dynamic changes in user preferences and the social relationships of users. Firstly, it uses a gated recurrent neural network to model user project interaction information and mine user project preference features. Then, based on the user correlation matrix, the attention mechanism is used to learn the social influence preference features of the target user. Finally, Top K recommendations for the user are provided by combining the user's project preference features with their social influence preference features. First, in terms of dynamic changes of user preferences, to better learn the changes of user preferences, since Gated Recurrent Unit (GRU) can effectively process timing information, it is applied to the data modeling of user project interaction sequence, and a user preference model based on interaction sequence is proposed^20–22^. GRU can achieve the goal of mining users' project preference features by efficiently recalling pertinent inputs using updating and resetting gates. It sorts user interaction items based on the order of interaction time, where $u$ represents the user and $S_{u}$ represents the interaction sequence of $u$. The expression is shown in Eq. (1).

1 $$S(u) = \left\{ {v_{1}^{u} ,v_{2}^{u} , \cdots ,v_{N}^{u} } \right\}.$$

**Figure 1:**
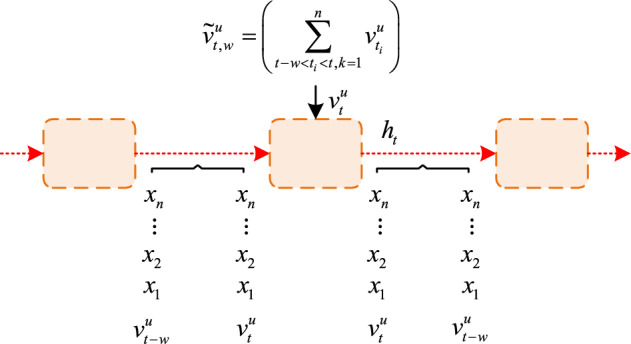
A dynamic preference model for user interaction sequences based on GRU.

In Eq. (1), it sets the $t$-th interaction item in $S_{u}$ to $v_{t}^{u}$ and sets the maximum retrieved value to $N$. The topic of each project is analyzed through the probabilistic topic model (LDA) to obtain the corresponding topic distribution $v_{t} = [\theta_{1} ,\theta_{2} , \cdots ,\theta_{n} ]$. In $v_{t}^{{}}$, it sets the probability under its $i$-th theme to $\theta_{i}^{{}}$. It sets the interval window to $w$. In GRU, it introduces a $w$ to learn about the periodic changes in user preferences. The user interaction sequence dynamic preference model based on GRU is shown in Fig. 1.

In Fig. 1, $h_{t}$ represents the hidden state, which consists of the interaction sequence information of $u$ and the topic preference in $w$. It sets the topic preference in $w$ to $\tilde{v}_{t,w}^{u}$. $x$ represents input; $n$ represents the quantity. In GRU, the interaction sequence of input $u$, the candidate $h_{t}$ for GRU at time $t$, which is also the mathematical expression of $\tilde{h}_{t}$, is shown in Eq. (2).

2 $$\tilde{h}_{t} = \tanh \left( {(v_{t}^{u} W_{v} + \left( {r_{t} \odot h_{t - 1} } \right)W_{h} + b_{h} } \right).$$

In Eq. (2), it sets the reset door to $r_{t}$; The hidden state at time $t - 1$ is represented as $h_{t - 1}$; $W_{v}$,$W_{h}$ represents the weight matrix, with the bias term set to $b_{h}$ and the element multiplier set to $\odot$. To obtain the main preference features of $u$ within $w$, $\tilde{v}_{t,w}^{u}$ is added as the project topic preference vector of $u$ within $w$. And each item of $\tilde{v}_{t,w}^{u}$ is accumulated by all project topics within $w$, so the topic distribution of $u$ interactive projects within $w$ is significantly represented, thus obtaining the main preference characteristics of $u$ within $w$. From this, it can be concluded that the mathematical expressions of $\tilde{h}_{t}$ and $h_{t}$ at time $t$ are shown in Eq. (3).

3 $$\left\{ \begin{gathered} \tilde{h}_{t} = \tanh \left( {(v_{t}^{u} \odot \tilde{v}_{t,w}^{u} )W_{v} + \left( {r_{t} \odot h_{t - 1} } \right)W_{h} + b_{h} } \right) \hfill \\ h_{t} = z_{t} \odot h_{t - 1} + (1 - z_{t} ) \odot \tilde{h}_{t} \hfill \\ \end{gathered} \right..$$

In Eq. (3), it sets the update door to $z_{t}$. Finally, the output at time $t$ is $c_{t} = \tanh (h_{t} )$. $c_{t}$ means the change in project preference of $u$ on $w$, and also represents the change in preference of the entire interaction sequence of $u$. It inputs it into the output layer, obtains the relevant output, and sets it as the $u$ project preference feature $o_{t}$. This feature consists of two parts, namely the interaction sequence information of $u$ and the preference within the local interval. The mathematical expression of $o_{t}$ is shown in Eq. (4).

4 $$o_{t} = \phi \left( {W_{o} c_{t} + b_{o} } \right).$$

**Figure 2:**
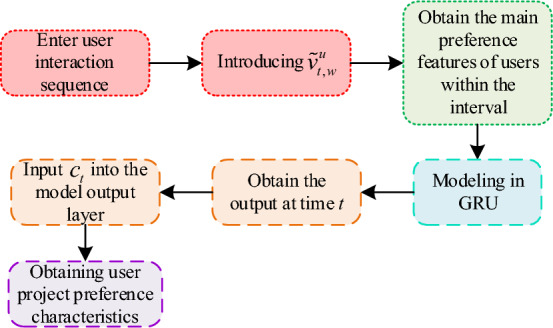
User preference feature extraction process.

In Eq. (4), it sets the ReLU function to $\phi \left( \cdot \right)$, $W_{o}$ represents the weight matrix, $I \in {\mathbb{R}}^{L}$, and sets the bias term to $b_{o}$. It summarizes the above user preference feature extraction and obtains the relevant extraction as shown in Fig. 2. Through the operation in Fig. 2, the required user preference features can be obtained, thus preparing for the following movie recommendation work.

### Preferential attention model based on social relationships

**Figure 3:**
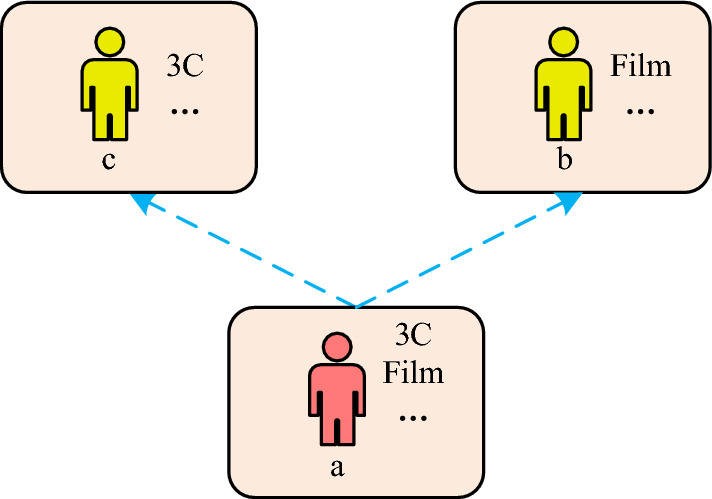
The impact of different users on user a.

When making movie recommendations, based on user preference features based on interaction sequences, the social relationships of users are taken into account. Then it improves data sparsity through user social networks and proposes a Preference Attention Model Based on Social Relationships (PASR), utilize attention mechanisms to mine user features in social networks as much as possible, thereby improving the predictive ability of the model. Firstly, it analyzes user social networks. Generally, users have varying social circles with limited shared preferences. And the impact of different users on user a is different, with user a as the center, forming a local social network as shown in Fig. 3.

**Figure 4:**
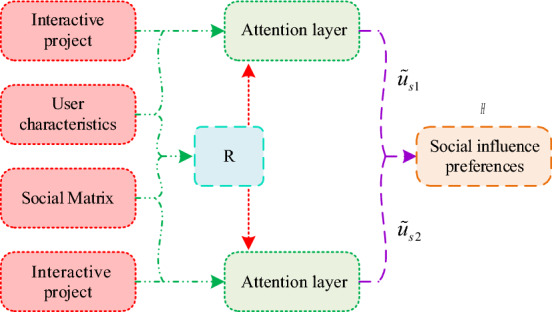
PASR model.

In Fig. 3, b represents user b; c represents user c; 3C represents a certain product; User b and user c are friends of user a; 3C products are a common concern for users c and a; Movies are a common concern for both user b and user a. When user a chooses 3C products, user c has the greatest influence on user a, and user a will ask for more opinions from user c. Similarly, if user a chooses a movie, then user b has the greatest impact on user a. Thus, in various projects and in the course of user interactions, accurately capturing the user's social influence preferences involves gauging the influence of different friends on the user's preferences and the degree to which the user is influenced by each friend. To obtain user social influence preferences, first, in a given user social network $G$, a deep network embedding model with aggregated proximity preservation (DNE-APP) is used to learn low dimensional embedding of network nodes^23^. The embedding method is the network embedding method^24^. This is to effectively preserve node content information and preserve network structure information. In the input $a_{i}$ of the pallet autoencoder, $i$ represents the node number; $a_{i}$ represents the characteristics of the corresponding node. After conducting relevant calculations, a network representation can be obtained. Subsequently, the low-dimensional feature representations of all nodes are output, and the obtained node feature representation matrix is applied to the PASR model, as shown in Fig. 4.

In Fig. 4, the model can output the user's social influence preferences, representing them as $H$. The user characteristics and social matrix are embedded by nodes; $\tilde{u}_{s1}$ represents the user's tendency towards friends, and $\tilde{u}_{s1}$ is obtained based on user interaction items $v_{j}$, $u_{u}$, and the correlation matrix $R$; It sets the social impact of friends on users to $\tilde{u}_{s2}$, and $\tilde{u}_{s2}$ is obtained based on $v_{j}$, $F_{u}$, and $R$. $R$ is derived from $u_{u}$ and $F_{u}$, and the vector in $R$ represents the social vector that affects user preferences. In the model, two attention layers are used to obtain $\tilde{u}_{s1}$ and $\tilde{u}_{s2}$, respectively. Different inputs to the attentional layer correspond to different output characteristics of the attentional layer. Taking into account $\tilde{u}_{s1}$ and $\tilde{u}_{s2}$, the model can obtain $H$. It is worth noting that in $F_{u}$, the number of interactive projects between each user's friend $f$ is greater than or equal to the number of projects within the target user $w$. Specifically, the mathematical expression of $R$ is shown in Eq. (5).

5 $$R = \phi \left( {F_{u} W_{rF} + \left[ {u_{u} ;w} \right]W_{ru} + b_{r} } \right).$$

In Eq. (5), $W_{ri}$ represents the weight matrix; $b_{r}$ represents the bias term; It sets the initial value of $w$ to 3 and determines the optimal size of $w$ based on experiments; $[;]$ represents splicing; Each feature in $F_{u}$ consists of two parts, namely the friend feature and its corresponding $N$. To obtain user preferences for their friends, input user features and $R$ in the attention layer. Calculate the user's attention score $a_{1}$ for friends when interacting with a certain item, $\alpha_{1} = \phi \left( {W_{au} u_{u} + W_{ar} R^{T} + W_{av} v_{j} + b_{{a_{1} }} } \right)$,$W_{au}$ represents the weight matrix, $b_{a1}$ represents the bias term. Input the calculated results into the softmax function to obtain the corresponding attention weight $\alpha_{1}$. According to this method, calculate the influence weight $\alpha_{2}$ of friends on users. The relevant formula is shown in Eq. (6).

6 $$\left\{ \begin{gathered} a_{2} = \phi \left( {W_{af} F_{u} + \left( {W_{au} u_{u} } \right)R^{T} + W_{av} v_{j} + b_{{a_{2} }} } \right) \hfill \\ \alpha_{2} = \frac{{\exp \left( {a_{2} } \right)}}{{\sum\nolimits_{i \in F} {\exp \left( {a_{2}^{i} } \right)} }} \hfill \\ \end{gathered} \right..$$

In Eq. (6), $a_{2}$ represents the user’s impact score on a certain aspect of a friend; $W_{af}$, $W_{au}$ and $W_{av}$ represent the weight matrix; $b_{a2}$ represents the bias term; $T$ represents transposition. It multiplies $\alpha_{1}$ and $u_{u}$ to obtain $\tilde{u}_{s1}$, and multiplies $\alpha_{2}$ with the user friend feature vector $f_{i}$ to obtain $\tilde{u}_{s2}$. Based on $\tilde{u}_{s1}$ and $\tilde{u}_{s2}$, it yields $H = (\tilde{u}_{s1} ,\tilde{u}_{s2} )$.

### Top-K recommendation algorithm and experimental design based on preference fusion

**Figure 5:**
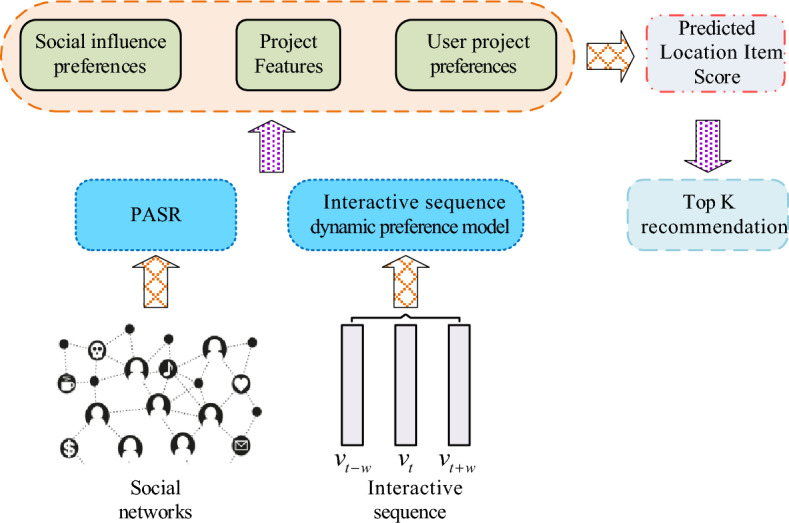
Schematic diagram of the algorithm.

Based on the user preference model and PASR model, a Top-K recommendation algorithm based on preference fusion, namely the UPSI algorithm, is proposed. K denotes the number of items with the highest recommended item score. Figure 5 displays the schematic diagram of this algorithm.

In Fig. 5, it is mainly divided into three parts: the PASR model, the interactive sequence dynamic preference model, and the UPSI algorithm. By using the PASR model and the interactive sequence dynamic preference model, social influence preferences, project features, and user project preferences are obtained. Subsequently, the UPSI algorithm applies this information for Top-K recommendation related work. By utilizing the model illustrated in Fig. 5, it is possible to determine users' social influence preferences and understand their project preferences, leading to increased recommendation accuracy. Specifically, first, use a topic model to obtain the distribution of relevant project topics; it obtains user project preferences through the distribution of user interaction sequence topics. By utilizing user social networks, a user's correlation matrix can be obtained. Based on this matrix, it determines the extent of user preference for friends and the societal influence of friends on users. On this basis, users' social influence preferences can be obtained. Subsequently, preference fusion is performed to obtain project preferences, social influence preferences, and project features, and corresponding fusion features are obtained. These fusion features are then input into a multi-layer perceptron (MLP) to compute a score for users who are interested in suggested items. The predicted score for a user $$u$$ clicking on item $$v$$ is $$\overset{\lower0.5em\hbox{$\smash{\scriptscriptstyle\frown}$}}{r}_{u,v}$$,$$\overset{\lower0.5em\hbox{$\smash{\scriptscriptstyle\frown}$}}{r}_{u,v} = W_{r}^{{}} y_{l} + b_{r}$$. During the calculation, the user will finally embed the representation $$u_{p} = [o_{t} ;u_{s} ]$$, fuse $$u_{p}$$ and $$v_{i}$$, and input the results into the multi-layer perceptron. In Top-K recommendation, based on the predicted scores of recommended items by users, the top K items are sorted from top to bottom, and the top K items are selected to form a recommendation list. The BPR algorithm, which is based on implicit feedback, has displayed promising results in ranking project recommendations. Therefore, this research applies it to the training of UPSI algorithm, introduces the regularization parameter $$\lambda$$, and constructs the related loss function.

It analyzes the effectiveness of the proposed UPSI algorithm and studies its performance in Top K recommendation tasks. In the experimental environment setting, Linux is selected as the operating system, Pycharm Professional Edition is used as the development tool, CDUA8.0 is used as the computing platform, and Python 1.0.0 is used as the deep learning framework. Table 1 presents essential information on the selected experimental dataset.

**Table 1:** Experimental Dataset Information.

Index	CiaoDVDs	Douban
Number of users	7375	129,490
Number of projects	99,746	58,541
Number of interactions	278,483	16,830,839
Social connections	111,781	1,692,952

Table 1 contains two datasets. They are the CiaoDVDs dataset and the Douban dataset, respectively. These datasets all contain user social relationships and can be used to validate social recommendation algorithms. The user's project rating range in both datasets is [1, 5]. The datasets consist of positive and negative samples, the former of which include scored items while the latter include unrated items. The experimental parameters divide two datasets into three subsets randomly, with proportions of 80%, 10%, and 10%. These subsets are subsequently treated as training sets, validation sets, and testing sets, respectively. It uses a Gaussian distribution with a mean of 0 and a variance of 0.01 to initialize the parameters of the UPSI algorithm. MLP chooses a three story tower structure, with the first level dimension consistent with the embedded dimension. Using the Adam gradient descent algorithm as the optimizer, the relevant parameter settings are shown in Table 2.

**Table 2:** Related parameter settings.

Parameter	Value situation
MLP first layer dimension	32
MLP second layer dimension	16
MLP third layer dimension	8
Learning rate	0.0001
Batch size	256
$w$	[5, 10, 15, 20, 25]
$\lambda$	[0.01,0.001,0.0001,0.00001]

In Table 2, the value range of $w$ is [5, 10, 15, 20, 25], and the value range of $\lambda$ is [0.01,0.001,0.0001,0.00001]. In the selection of comparison methods, combining matrix decomposition and social networks, the trust propagation mechanism is introduced into the recommendation system, and the algorithm is set as the SocialMF algorithm^25^; The recommendation system employs the NCF algorithm, which utilizes matrix decomposition and MLP to facilitate understanding the interaction between users and projects. ScAN algorithm, based on social networks and deep learning neural networks, is employed for project recommendations^26^. It chooses the CNSR algorithm, which combines the relationship between social network structure and user-project interactions, and utilizes relevant joint learning frameworks to perform social recommendations. It selects Hit Ratio (HR) to evaluate whether the test item appears in the top K items of the prediction recommendation list. If it appears, it means hit. The calculation formula for hit rate $HR$ is shown in Eq. (7).

7 $$HR = \frac{{\sum {Hits} }}{{\left| {TN} \right|}}.$$

In Eq. (7), among the recommended projects by each user, the number of projects classified as the test set is set to $\sum {Hits}$, and the comprehensive number of projects in the test set is set to $\left| {TN} \right|$. It selects Normalized Discounted Cumulative Gain (NDCG) to evaluate whether the test item appears in the top K items of the recommendation list and analyze the accuracy of the recommendation list arrangement.

## Analysis of the application results of UPSI algorithm in movie recommendation

**Figure 6:**
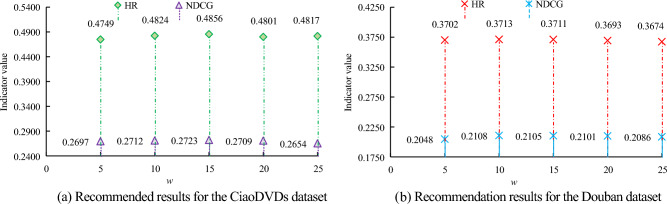
The effect of different interval window values on algorithm performance.

In movie recommendation, the performance of the UPSI algorithm is analyzed, and the value range of $w$ is [5, 10, 15, 20, 25]; The experimental dataset includes the CiaoDVDs dataset and the Douban dataset; The evaluation indicators are HR and NDCG. The performance of the UPSI algorithm under different interval window $w$ values is studied, and the relevant results are shown in Fig. 6.

**Figure 7:**
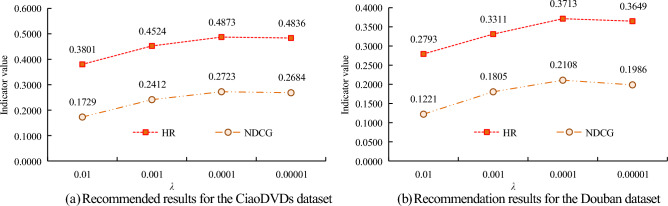
Influence of different regularization parameters on algorithm performance.

Figure 6 shows the algorithm results for different $w$ values in the CiaoDVDs dataset in subgraph (a); Fig. 6 subgraph (b) shows the algorithm results for different $w$ values on the Douban dataset. In subgraph (a) of Fig. 6, with different $w$ values, the HR and NDCG values of the algorithm are different. As the $w$ value increases, the HR value of the algorithm gradually increases and then decreases, and then increases again. The NDCG value gradually increases and then decreases; And under the same $w$-value, the algorithm's HR value is greater than the NDCG value. When the $w$ value is 5, the HR value of the algorithm is 0.4749, which is 0.0075 less than the $w$ value of 10. The HR value of the latter is 0.4824, while the NDCG value of the algorithm is 0.2697; When the $w$ value is 15, the corresponding HR value of the algorithm is 0.4856, which is higher than when $w$ takes other values; The NDCG value of the algorithm is the highest at 0.2723; When the $w$ value is 20 and 25, the corresponding HR values of the algorithm are 0.4801 and 0.4817, respectively, while when the $w$ value is 25, the minimum NDCG value of the algorithm is 0.2654. In subgraph (b) of Fig. 6, as the $w$-value increases, the HR and NDCG values of the algorithm first increase and then decrease. When the $w$ value is 10, the HR value and NDCG value of the algorithm are both the highest, with values of 0.3713 and 0.2108, respectively; When the $w$ value is 15, the HR value of the algorithm is 0.3711, which is smaller than when the $w$ value is 10; When the $w$ value is 25, the HR value and NDCG value of the algorithm are both the smallest, 0.3674 and 0.2086, respectively. According to the relevant results of the two subgraphs in Fig. 6, it demonstrates that in the CiaoDVDs dataset, the model performs best with a $w$ value of 15, while in the Douban dataset, the optimal value for $w$ value is 10. The performance of the algorithm under different regularization parameter $\lambda$ is studied, and the results are shown in Fig. 7.

**Figure 8:**
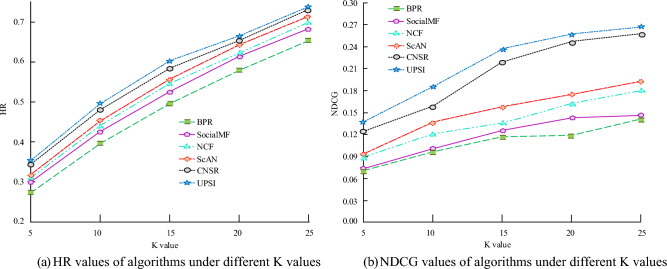
Performance of different algorithms under different K values.

In sub graph (a) of Fig. 7, under different regularization parameters $\lambda$, the HR value and NDCG value of the algorithm are different. When $\lambda$ value increases, the HR value and NDCG value of the algorithm first increase and then decrease. When the $\lambda$ value is 0.01, the HR value and NDCG value of the algorithm are both the smallest, 0.3801 and 0.1729, respectively; When the $\lambda$ value is 0.001, the HR value of the algorithm is 0.4524, which is 0.0312 less than the $\lambda$ value of 0.00001; The HR value of the latter is 0.4836; When the $\lambda$ value is 0.0001, the HR value and NDCG value of the algorithm are both the highest, 0.4873 and 0.2723, respectively. In sub graph (b) of Fig. 7, under different regularization parameters $\lambda$, the change trend of algorithm HR value and NDCG value is the same as that in sub graph (a) of Fig. 7. When the $\lambda$ value is 0.0001, the HR value and NDCG value of the algorithm reach their maximum. When the $\lambda$ value is 0.001, the HR value of the algorithm is 0.3311, which is 0.0518 greater than the $\lambda$ value of 0.01; When the $\lambda$ value is 0.0001, the NDCG value of the algorithm is 0.1986. The two subgraphs in Fig. 7 demonstrate that the optimal value of the regularization parameter $\lambda$ is 0.0001. In the Top K recommendation, the CiaoDVDs dataset is selected to analyze the overall performance of the UPSI algorithm. BPR, SocialMF, NCF, Scan, and CNSR are selected as comparison methods to study the performance of different algorithms under different K values. The results are shown in Fig. 8.

**Figure 9:**
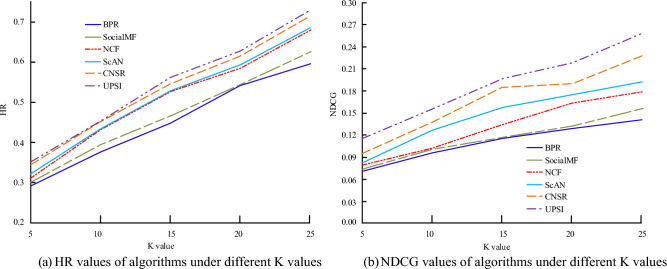
Recommendation results under the Douban dataset.

In subgraph (a) of Fig. 8, as the K value increases, the results of the six algorithms gradually increase. The UPSI algorithm's line is located above the others, while the BPR algorithm's line is located at the bottom. When the K value is 15, the HR values of the UPSI algorithm and Scan algorithm are 0.607 and 0.566, respectively; When the K value is 20, the HR value of the NCF algorithm is 0.624, which is 0.671 less than the UPSI algorithm; For K values of 25, the HR values of the five algorithms are all the highest, and the NCF algorithm has an HR value of 0.739. In subgraph (b) of Fig. 8, with the increase of K value, there are certain differences in the trend of NDCG values among the six algorithms, showing an overall upward trend; under the same K value, the UPSI algorithm has the highest NDCG value, followed by the CNSR algorithm, and the BPR algorithm has the lowest NDCG value. When the K value is 15, the NDCG value of the BPR algorithm is 0.118, while the NDCG values of the UPSI algorithm and the Scan algorithm are 0.236 and 0.158, respectively; When the K value is 25, the NDCG value of the UPSI algorithm is the highest, at 0.267, which is 0.120 higher than the SocialMF algorithm, and the NDCG value of the latter is 0.147. The impact of the K value on the algorithm's recommendation results is evident. The UPSI algorithm displays superior recommendation results and performance compared to other algorithms. Other conditions remain unchanged, and the performance of different K values and algorithms under the analysis of the Douban dataset is shown in Fig. 9.

**Figure 10:**
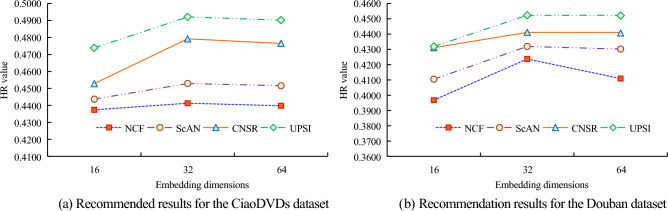
Recommendation performance of algorithms under different embedding dimensions.

In subgraph (a) of Fig. 9, as the K value increases, the results of the six algorithms gradually increase, and the line where the UPSI algorithm is located is always above the other algorithms. When the K value is 20, the HR value of UPSI algorithm is 0.637, while the HR value of NCF algorithm is 0.585; When the K value is 25, the HR value of the NCF algorithm is 0.731. In subgraph (b) of Fig. 9, as the K value increases, the NDCG values of the six algorithms generally show an upward trend. Under the same K value, the NDCG value of the UPSI algorithm is the highest. When the K value is 5, the NDCG value of the BPR algorithm is 0.069; When the K value is 25, the NDCG value of the UPSI algorithm is the highest, at 0.256, which is 0.101 larger than the SocialMF algorithm. Based on the data shown in Fig. 9, it is evident that choosing a suitable K value enhances the algorithm's recommendation performance. Furthermore, the UPSI algorithm outperforms the others in terms of recommendation results and performance. Figure 10 displays the analysis of the recommendation performance of four algorithms at varying embedding dimensions.

**Figure 11:**
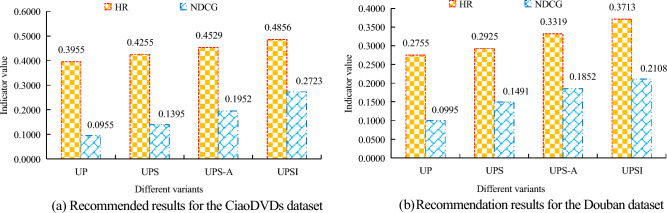
Comparison results of different variants.

In subgraph (a) of Fig. 10, different embedding dimensions result in different HR values for the algorithm. The HR value for the UPSI algorithm is 0.4921 when the embedding dimension equals 32, which is 0.0129 greater than the value for the CNSR algorithm. The HR value of the algorithm in this embedding dimension is greater than the other two embedding dimensions. In subgraph (b) of Fig. 10, the trend of HR values for the four algorithms under different embedding dimensions is similar to that in subgraph (a) of Fig. 10. The HR value of the UPSI algorithm at an embedding dimension of 64 is 0.4521, which is 0.0002 lower than that at 32. Notably, the algorithm with an embedding dimension of 32 exhibits the highest HR value compared to other dimensions, particularly the UPSI algorithm. From this, it can be seen that the optimal value for embedding dimensions is 32, which also indicates the better performance of the UPSI algorithm. It conducts relevant ablation experiments and sets only the interaction sequence between the user and the project to UP. Based on UP and combined with user social relationships, only represent the social impact of users through network embedding, and set this part as UPS. The meaning of UPS-A is to represent the social impact of users through a uniform distribution strategy based on UPS. Its analysis of the performance of different variants and UPSI in different datasets is shown in Fig. 11.

In subgraph (a) of Fig. 11, the performance of UP is the worst, with its corresponding HR and NDCG values smaller than other variants, with an HR value of 0.3955 for UP; In the HR indicator, the maximum value of UPSI is 0.4856, which is 0.0333 higher than UPS-A, 0.0601 higher than UPS, and 0.0901 higher than UP. For the NDCG indicator, the highest value is for UPSI, at 0.2723. By comparing the relevant indicator values of different variants in subgraph (b) of Fig. 11, it can also be observed that the HR and NDCG values under UPSI are the highest. This suggests that UPSI outperforms the others and is more suitable for the display scenario. To further verify the superiority of the UPSI algorithm, a recommended algorithm based on Stack denoising automatic encoder (SDAE) was selected for comparison, and the CiaoDVDs dataset was selected. The relevant results are shown in Table 3^27^.

**Table 3:** Comparison results of two methods.

Evaluating indicator	UPSI algorithm	SDAE algorithm
HR value	0.4913	0.4145
NDCG value	0.2674	0.1647

In Table 3, there are differences in the HR and NDCG values between the two algorithms. The UPSI algorithm outperforms the SDAE algorithm as it has higher HR and NDCG values. Among them, the HR value of the UPSI algorithm is 0.4913, which is 0.0768 higher than the SDAE algorithm, while the latter is 0.4145. These results indicate that the UPSI algorithm has better recommendation performance and higher accuracy.

In the process of movie recommendation, considering the dynamic changes in user preferences and social relationships among users is beneficial for improving the accuracy of recommendations. Some scholars face the problem of real-time interest point recommendation, considering the changes in user real-time preferences, and combining short-term and long-term memory networks to obtain corresponding real-time preference mining models. They mine user preferences from both long-term and short-term preferences. Filter through designed categories to reduce search space and improve recommendation effectiveness. The results show that the proposed method has a high recall rate^28^. Some scholars, in order to improve the effectiveness of tourism recommendations, take into account the social relationships of users and obtain a social mixed recommendation system for tourist attractions based on a social business environment. This system provides tourists with a personalized list of attractions. Experimental verification indicates that the proposed method is effective in generating recommendations^29^. From this, it can be seen that dynamic changes in user preferences and social relationships can greatly affect recommendation effectiveness. Therefore, relevant recommendation research must consider these factors to enhance recommendation efficacy.

## Conclusion

To improve the effectiveness of movie recommendations and fully consider the correlation between user preferences, research is conducted from the perspective of user preferences and social impact. Then, based on the interaction sequence, this study constructs a user preference model and extracts user preference features. Finally, the study constructs a PASR model using the attention mechanism to extract user social influence preferences, develops a social model based on the attention model, known as the UPSI algorithm, and performs experimental analyses. The results show that the interval window $w$ can affect the performance of the UPSI algorithm. In the CiaoDVDs dataset, the optimal value of $w$ is 15; In the Douban dataset, the optimal value for $w$ is 10. The appropriate regularization parameter $\lambda$ is conducive to improving the performance of the algorithm, and the optimal value of $\lambda$ is 0.0001. When the $\lambda$ value is 0.0001, the HR value and NDCG value of the UPSI algorithm are both the highest, 0.4873 and 0.2723, respectively. In the Top K recommendation, the UPSI algorithm has the best results compared to other algorithms. In the CiaoDVDs dataset, when the K value is 25, the UPSI algorithm has the highest HR and NDCG values, with values of 0.739 and 0.267, respectively. The embedding dimension value can affect the performance of recommendation algorithms. When the embedding dimension is 32, the algorithm performs best, especially the UPSI algorithm. At this point, the HR value of the UPSI algorithm is 0.4921, which exceeds the HR value of the CNSR algorithm by 0.0129. Compared to other variants, UPSI performs the best, with a maximum HR value of 0.4856 in the CiaoDVDs dataset. This is 0.0333 higher than UPS-A. From this, it can be seen that the movie recommendation algorithm proposed in the study has a good application effect. In the future, we can use the natural language processing method to analyze user comments from the perspective of the user’s rating data of the project. Ultimately, user preference features can be obtained.

## Data Availability

All data generated or analysed during this study are included in this published article.
